# Flat dose regimen of toripalimab based on model-informed drug development approach

**DOI:** 10.3389/fphar.2022.1069818

**Published:** 2023-01-13

**Authors:** Lili Li, Jianye Qu, Ming Song, Qun Zhao, Yonghua Yang, Xi Tan, Yanyan Hu, Jing Li, Yunfei Lin, Hui Feng, Sheng Yao, Patricia Keegan, Meixia Chen

**Affiliations:** ^1^ Shanghai Junshi Biosciences, Shanghai, China; ^2^ TopAlliance Biosciences, Rockville, MD, United States

**Keywords:** flat dose, toripalimab, model-informed drug development approach, population pharmacokinetics, exposure-response analysis

## Abstract

**Introduction:** Flat dosing regimen has recently been approved for programmed death receptor-1 (PD-1) inhibitors including toripalimab, nivolumab and pembrolizumab. The objective of this study is to provide pharmacological evidence for a flat dosing regimen of toripalimab by assessing the efficacy and safety profile of a 240 mg Q3W flat dose relative to the currently approved 3 mg/kg Q2W.

**Methods:** A population pharmacokinetic (PopPK) model was established based on 1,014 evaluable patients in 13 clinical studies. The exposure-objective response rate (ORR, n = 234) and exposure-safety (n = 152) analyses were performed by logistic regression. Three safety endpoints including grade ≥ 3 adverse events (AEs), treatment-related grade ≥ 3 AEs, and AEs leading to study drug discontinuation were evaluated. Progression-free survival (PFS, n = 234) was evaluated using a Cox proportional hazard model with the Kaplan-Meier survival curve.

**Results:** The PK profiles of toripalimab are best described by a two-compartment model with time-varying clearance characterized by a sigmoidal maximum effect (E_max_) function. Simulations for the first dose and steady-state exposures for the 240 mg Q3W dosing regimen were comparable to those for the 3 mg/kg Q2W dosing regimen with 95% exposure coverage ranging from 88% to 96%. The exposure-safety analysis showed that the probability of an adverse event occurring did not increase with increases in toripalimab exposure. A flat exposure-response relationship for ORR was identified. The Kaplan–Meier survival curve showed that exposure was a predictor for PFS; however, no difference in treatment benefit was demonstrated across exposure quantiles using a Cox proportional hazard model.

**Discussion:** This study revealed that toripalimab exposure of 240 mg Q3W dosing regimen was comparable to 3 mg/kg Q2W dosing regimen. The safety and efficacy E-R results of 240 mg Q3W is flat. Hence, the 240 mg Q3W dosing regimen is determined to be a preferred therapeutic dosage for toripalimab due to the convenience of flat dose.

## 1 Introduction

Toripalimab is a humanized monoclonal antibody for programmed death receptor-1 (PD-1); it can bind to PD-1 and prevent the binding of PD-1 with programmed death-ligand 1 (PD-L1) and programmed death-ligand 2 (PD-L2) (Patsoukis et al., 2020). Toripalimab was developed by Shanghai Junshi Bioscience Co., Ltd. for cancer treatment (Keam, 2019). In June 2021, the Chinese patent gold award was granted to this company for this remarkable discovery. In China, toripalimab (240 mg Q3W) in combination with chemotherapy was approved as the first-line treatment for unresectable locally advanced or metastatic non-squamous non-small cell lung cancer (NSCLC) with no EGFR or ALK genomic tumor aberrations (Wang Z. et al., 2022), recurrent or metastatic nasopharyngeal carcinoma (NPC) (Mai et al., 2021) and advanced esophageal squamous cell carcinoma (ESCC) (Wang Z. X. et al., 2022). Toripalimab monotherapy (3 mg/kg Q2W) was approved as the second-line treatment for unresectable or metastatic melanoma (Tang et al., 2020) and locally advanced or metastatic urothelial carcinoma (UC) (Sheng et al., 2022) and as the third-line treatment for recurrent or metastatic NPC (Wang et al., 2021). In addition, the US Food and Drug Administration (FDA, 2022a) designated toripalimab as a breakthrough therapy for first-line and second/third-line treatment of recurrent or metastatic NPC. Thus far, more than 30 clinical studies have been conducted in patients with dozens of indications in Asia, North America, and Europe. The findings of phase II and/or phase III studies have been presented at meetings held by American Society of Clinical Oncology (Xu et al., 2021a; Wang J. et al., 2022), Chinese Society of Clinical Oncology, European Society for Medical Oncology (Xu et al., 2021b), American Association for Cancer Research(Mai et al., 2022), and World Conference on Lung Cancer (Zhang et al., 2019).

Weight-based dosing is typically used for monoclonal antibody (mAb) drugs to reduce inter-subject variability in treatment efficacy (Bai et al., 2012). However, weight-based dosing is still under debate because there is little to no clinical evidence supporting that it significantly reduces inter-subject variability in terms of drug exposure. Drug exposure outcome can be impacted and/or convoluted by many environmental factors, such as concomitant medication and herbal supplements, and intrinsic factors, such as age, sex, race, body weight, and other comorbid diseases (Bai et al., 2012; Hendrikx et al., 2017). Body weight alone may not be able to explain the overall inter-subject variability of drug exposure. Additionally, mAbs show several unique characteristics in drug tissue distribution and drug elimination. Studies show that drug distribution is primarily in blood plasma and extracellular fluids, which are not directly proportional to body weight (Hendrikx et al., 2017). The mAbs are eliminated *via* proteolytic catabolism, which is a non-specific immunoglobulin elimination pathway, and intracellular degradation after binding to their targets (Hendrikx et al., 2017). The elimination rate of antibodies from tissues is primarily determined by the convective elimination clearance and by the rates of antibody catabolism within tissues, which are also not correlated with body weight. Studies have shown that weight-based dosing regimens do not significantly reduce inter-subject variability in PK of anti-PD1 antibodies, such as nivolumab (Zhao et al., 2017) and pembrolizumab (Freshwater et al., 2017). Physicians are increasingly preferring flat dosing for clinical convenience and reducing active drug ingredient waste and medication errors. Consequently, alternative dosing strategies, such as flat dosing, should be considered in dose escalation and dose selection during drug development programs.

Notably, a weight-based dosing regimen of 0.3–10 mg/kg Q2W and flat dose regimens of 80– 480 mg Q2W and 240 mg Q3W were both explored in toripalimab phase I dose escalation studies. No dose-limiting toxicity was observed in phase I studies (Wei et al., 2020), indicating the considerable therapeutic window of toripalimab. The recommended phase II dose (RP2D) of toripalimab relied on PK assessment of phase I and the ability of toripalimab to sustain its serum concentration required to achieve full receptor occupancy. *In vitro* experiments demonstrated that PD-1 receptors on the surface of T cells were saturated with a toripalimab plasma concentration >20 nM or 3 μg/mL. To ensure full receptor occupancy in tumor microenvironment, 3 mg/kg Q2W and 240 mg Q3W dosing regimens were recommended to achieve the target trough concentration (20 μg/mL) of toripalimab.

With the advances in scientific methods, novel modeling and simulation approaches have been developed to facilitate medical product discovery. Model-informed drug development (MIDD) approach is to establish quantitative models based on preclinical and clinical data to assist decision-making for drug development process. The top three areas for the MIDD program identified by the FDA are dose estimation or selection, clinical trial simulation, and safety evaluation (Wang et al., 2020). The FDA approved nivolumab 480 mg every 4 weeks (Q4W) as an alternative dosing regimen to nivolumab 240 mg Q2W based on the results of a MIDD analysis (Bi et al., 2019). Toripalimab was initially approved with a weight-based dosing regimen of 3 mg/kg Q2W intravenously (Keam, 2019; Wang Z. X. et al., 2022) and with a flat dose of 240 mg Q3W administered in combination with chemotherapy. Flat dose regimens that can be used alternatively would offer greater convenience to patients in their cancer treatment, particularly for combination regimens with diverse dosing requirements. The goal of this study was to provide pharmacokinetic evidence supporting flat dosage of toripalimab in antitumor therapy.

## Methods

### Dataset

A toripalimab PopPK model was developed based on data of 1,014 patients derived from 13 clinical studies on various cancer types, including advanced melanoma, UC, gastric cancer (GC), ESCC, NPC, non-small-cell lung cancer, head and neck squamous cell carcinoma, sarcoma, and lymphoma. Toripalimab concentrations were determined using validated assays. During PK analysis, concentrations below the limit of quantitation of the assays at various time points were kept in the analysis dataset and flagged accordingly. If >10% of data was below the lower limit of qualification for any single analyte, then likelihood-based methods of imputation were considered (e.g., M3 likelihood imputation). Data were collected from patients who received toripalimab as monotherapy (CT 1–4, CT 5 cohort 1–4 and CT 6–9, CT 12, CT 14, TAB001-01) or in combination with platinum-based chemotherapy (CT 5 cohort 5–8 and JUPITER-02). The PK of the scheduled single and multiple toripalimab dosages of 0.3–10 mg/kg Q2W and scheduled flat doses at 80—480 mg Q2W and 240 mg Q3W was investigated. Study descriptions, study numbers, and relevant information are summarized in Supplementary Table S1.

Toripalimab safety exposure-response (E-R) analysis included 152 NPC patients who had both PK information and AEs from the JUPITER-02 study. The efficacy E-R objective response rate (ORR) analysis was performed on 234 patients who had data available for both PK and best overall response in the JUPITER-02 study. The efficacy E-R progression-free survival (PFS) analysis was performed on 234 NPC patients who had information available for both PK and PFS in the JUPITER-02 combination study.

### PopPK analysis

#### Modeling approach

A non-linear mixed-effects modeling (NONMEM) approach was used in PopPK analysis. Initially, a base model was developed to describe the PK of toripalimab without taking into consideration covariate effects. The development of this base model included the development of a structural model, interindividual variability (IIV) model, and residual error model. Structural model development included the assessment of temporal changes in toripalimab clearance (CL). The sigmoidal E_max_ functional form was used to describe time-dependent CL. The base model with a sigmoidal E_max_ functional form was compared with the base model with constant CL. The function form to describe the CL time dependency included the sigmoidal E_max_: E_max =_ $\exp\left(\frac{E_{\max}*T^{Gamma}}{{T_{50}}^{Gamma}+T^{Gamma}}\right)$

The IIV on the E_max_ parameter is expressed as follows: $E_{\max_{i}}{=E}_{\max_{TV}}+\eta_{E_{\max}}$ ,

where E_maxTV_ represents the population (typical value) estimate of the maximal change in CL and η_Emax_ represents the IIV of E_max_ with mean 0 and variance ω_Emax_^2^. The T_50_ parameter represents the time at which the change in CL is 50% of E_max_, and γ represents the sigmoidicity of the relationship with time after the first dose.

#### Covariate identification

The evaluation of the impact of covariates on PopPK focused on the most clinically relevant covariates. The following continuous covariates were included: body weight, age, baseline albumin (BALB), baseline lactate dehydrogenase (BLDH), baseline alkaline phosphatase, baseline aspartate aminotransferase (BAST), baseline alanine aminotransferase (BALT), baseline total bilirubin (BILI), baseline tumor burden (TUBURBL; sum of the longest diameters of all target lesions), and creatinine clearance (CRCL). The following categorical covariates were included: sex, race, the anti-drug antibody (ADA) status, tumor type, the Eastern Cooperative Oncology Group (ECOG) performance status, and combination treatment (COMBOTRT). Kidney impairment at baseline (KIDIMBLN) and liver impairment at baseline (LVRIMBLN) were tested as additional sensitivity covariates. Baseline renal function based on the estimated renal function was calculated using the Cockcroft–Gault formula from serum creatinine, and baseline liver dysfunction was based on the grade as per the National Cancer Institute common terminology criteria for adverse events version 5.0. Covariates were selected using a forward addition process followed by backward elimination. The likelihood ratio test was used to evaluate the significance of incorporating flat effects into or removing flat effects from the population model on the basis of the significance levels that were set *a priori*. For forward addition and backward elimination, significance levels of 0.01 and 0.001 (*p*-value) were used, respectively.

All continuous covariates were incorporated into the population model using a scaled structure on the basis of the median value of the covariate in the population. This approach ensured that covariate effects were relative to a subject in the middle of the population distribution for that covariate. All categorical covariates were initially incorporated into the population model using a proportional structure with either the most common level of the covariate being the reference or by choosing a level specific to the analysis (e.g., ECOG 0 vs. ECOG not 0). This approach ensured that categorical covariate effects were relative to a reference group or category. The mathematical structures of the covariate models are shown below:

Continuous $P_{ki}=\theta_{k}*{\left(\frac{X_{ij}}{M\left(X_{j}\right)}\right)}^{\theta_{j}}$ Categorical ${\;P}_{ki}=\theta_{k}*{\left(1+\theta_{j}\right)}^{X_{ij}}$

where P_ki_ is the population estimate of the parameter P_k_ for the subject i, X_ij_ is the value of the continuous covariate X_j_ for the subject i or an indicator variable for the subject i for the categorical covariate X_j_ with a value of 1 for the non-reference category and 0 for the reference category, M(X_j_) is the median of the covariate X_j_ in the analysis dataset, θ_k_ is the typical value of the parameter P_k_, and θ_j_ is a coefficient that reflects the effect of the covariate X_j_ on the parameter.

At the end of covariate testing, alternative variance–covariance structures for Ω were evaluated, including partial and full block structures. Such a structure was deemed suitable as it provided a statistically significant (*P* < 0.001) improvement in the model objective function and improved model stability as measured by the condition number and/or indicated by a successful covariance step.

#### Model qualification

The final PopPK model was qualified using goodness-of-fit, bootstrap resampling, and a visual predictive check (VPC). A non-parametric bootstrap analysis performed for up to 1,000 replicates of the dataset was conducted to evaluate the stability of the final model and estimate confidence intervals (CIs) for the model parameters. The final model was repeatedly fitted to bootstrap replicates of the dataset. Notably, CIs were calculated on the basis of the distribution of the parameter estimates from the bootstrap runs.

VPCs were performed with prediction correction and used to evaluate the predictive ability of the final model. Plots of observed data distributions were compared to simulated distributions to demonstrate the model’s ability to adequately predict the data on which the model was based. VPCs were based on 500 simulations and were stratified by covariates of potential interest.

#### *Post-hoc* exposure estimates from the final PopPK model

The final PopPK model of toripalimab was used to obtain individual *post-hoc* estimates of PK model parameters. For each patient with measurable toripalimab concentrations available, PK exposure metrics [average serum concentration–time curve over the dosing interval (C_avg_), maximum serum concentration (C_max_), and trough serum concentration (C_trough_)] were estimated on the basis of *post-hoc* compartmental PK parameters for the first dose and at steady state (SS) for 3 mg/kg Q2W and 240 mg Q3W. The CL parameter at SS for a patient is calculated on the basis of covariate effects on CL and the E_max_ parameter only: ${CL}_{ss,i}{=CL}_{i}\;*\;\exp\left(E_{\max_{i}}\right)\;*\;{CLCOV}_{i}$ ,

where CL_ss,i_ represents the steady state CL of patient i, CLCOV_i_ represents the multiplicative covariate effects on CL, and E_maxi_ represents the individual maximal change in CL.

## E-R analysis

### E-R safety and E-R efficacy (ORR) logistic regression model analysis

#### Logistic regression model approach

E-R modeling is a sequential two-step PK/pharmacodynamic fitting process in which the actual dosing histories were used in the final PopPK model to derive individual PK parameters and average concentration (C_ave_). These outputs were then used to develop a logistic regression model.

Logistic regressions were performed to describe the relationship of toripalimab exposure with the safety endpoints [grade ≥ 3 adverse events (AEs), treatment-related grade ≥ 3 AEs, and AEs leading to study drug discontinuation] and the probability of ORR (efficacy endpoint defined by the best overall response of confirmed complete or partial response as determined by an independent review committee according to the Response Evaluation Criteria in Solid Tumors version 1.1) after adjusting for the effect of other significant covariates.

#### Predictor identification

The E-R model predictors fit for an efficacy endpoint (ORR) included terms for the full set of explanatory variables in the PopPK dataset. Then, a stepwise backward elimination procedure was applied using the step Akaike information criterion function from the Modern Applied Statistics with S package for R (4th edition). After the final backward elimination procedure, the resulting model was further assessed for biological plausibility. In this step, model predictors that were retained throughout the statistical evaluation but did not have a plausible biological interpretation were optionally deleted from the model. The exposure predictor was kept in the final model regardless of its statistical significance.

#### Logistic regression model qualification

Model evaluation followed similar general principles as described in the PopPK analysis. For logistic regression, model discriminatory performance was assessed by VPCs.

### E-R efficacy (PFS) analysis

The Kaplan–Meier (KM) survival curves for PFS were generated by C_ave_ quantiles. The *p* values were calculated from the log-rank test. To isolate the impact of exposure from that of the other predictors, KM survival curves were plotted for control and treatment groups. If a predictor was identified as significant in both control and treatment KM plots (*p* < 0.001), it was included in the Cox proportional hazard model.

### Software

The non-linear mixed-effects modeling software NONMEM^®^ (version 7.4.3; ICON, Hanover, MD, US), a non-linear mixed-effects analysis software package, was used for PopPK modeling. Xpose and Perl-speaks NONMEM (Department of Pharmacy, Uppsala University, Uppsala, Sweden) were also used for model diagnostics and facilitation of NONMEM tasks like covariate testing. R (versions 3.6.3 and 4.0.4) was used for graphical analysis, model diagnostics, and statistical summaries during PopPK model development. E-R analysis and *post-hoc* exposure from the PopPK model was estimated using R.

## Results

### Population PK analysis of toripalimab

#### Analysis dataset

The PK analysis dataset included 10,430 PK observation records from 1,014 patients in 13 clinical studies. Supplementary Table S1 shows pertinent details regarding the subject population, dosing regimen, and patient number and PK sample number for each study.

#### PopPK model development

One- and two-compartment structural models with first-order elimination were evaluated by fitting the dataset described in methods. The two-compartment model was preferred over the one-compartment model due to the lower objective function value (OFV). The CL of toripalimab was determined to be time-varying because the OFV was 1,233 units higher in the model with constant CL than in the model with time-varying CL. Therefore, the base model was a two-compartment structural model with zero-order IV infusion and time-varying CL characterized by a sigmoidal E_max_ (maximal change in CL from baseline) function. This final structure model was consistent with that of several anti-PD-1 antibodies (2022b; 2022a). The proportional error model was selected over comparison with additive and combined residual error models. IIV was evaluated for all parameters, but the model was more stable when IIV were evaluated on the basis of CL and the central volume of distribution (V_1_).

Using the base PopPK model, a covariate analysis was performed. To identify the covariates that were likely to significantly affect the CL and V_1_ of toripalimab, the influence of continuous and categorical covariates as specified in the methods section was included in the stepwise covariate model (SCM). Significant covariates of CL included body weight, albumin levels (ALB), LDH, CRCL, sex, and ADA-positive status. Significant covariates of V_1_ included race and body weight.

To obtain the final model, model refinements after sensitivity analysis were performed with the full covariate model based on the SCM result. During model refinement, tumor type was removed from the model. In addition, the effects of ALB on V_1_, KIDIMBLN on CL, and LVRIMBLN on CL were not statistically significant covariates in the model as per sensitivity analyses.

The parameter estimates for the final PopPK model are presented in Table 1. The time-varying CL results showed that, on average, CL decreases by approximately 31% compared to the baseline CL. In a typical patient, the time at which the change in CL is at 50% of E_max_ was approximately 65 days. This observation is consistent with the time-varying CL observed for other approved anti-PD-1 antibodies (2022b; 2022a). The percentage of the coefficient of variation (%CV) of IIV was 39% for E_max_, 31% for CL, and 27% for V_1_. The extent of shrinkage for IIV was <30% on E_max_ and CL and 38% on V_1_, indicating that the parameter estimates are reliable.

**TABLE 1:** Final PK model parameter along with 95% CIs obtained from bootstrapping runs.

Parameters	Estimates	%RSE	95% CI		Bootstrap median	Bootstrap 95% CI
E_maxTV_	−0.444	11	(−0.54, −0.35)		−0.43	(−0.54, −0.35)
T_50_ (h)	1,580	17	(1,051, 2,104)		1,615	(1,237, 3,000)
Gamma	1.32	14	(0.97, 1.68)		1.33	(0.98, 1.72)
CL_TV_ (mL/h)	14.9	2	(14.2, 15.6)		14.8	(14, 15.6)
CL_ADA_	0.191	24	(0.102, 0.280)		0.192	(0.109, 0.29)
CL_LDH_	0.161	13	(0.12, 0.20)		0.162	(0.12, 0.20)
CL_Female_	−0.19	11	(−0.23, −0.15)		−0.19	(−0.2, −0.15)
CL_Albumin_	−0.676	15	(−0.87, −0.49)		−0.69	(−0.89, −0.50)
CL_Weight_	0.097	65	(−.026, −0.22)		0.096	(−0.27, 0.23)
CL_CRCL_	0.226	17	(0.15, 0.30)		0.229	(0.14,0.30)
V_1_ (mL)	3,710	3	(3,511, 3,899)		3,707	(3541,3868)
V_1White Race_	−0.23	13	(−0.29, −0.17)		−0.23	(−0.29, −0.17)
V_1Other Race_	−0.327	10	(−0.39, −0.26)		−0.33	(−0.38, −0.25)
V_1Weight_	0.488	18	(0.31, 0.66)		0.5	(−0.32, −0.66)
Q_TV_ (mL/h)	36.5	73	(−16.05, 89.01)		30	(11.2, 85.4)
V_2TV_ (mL)	796	16	(549, 1,042)		866	(586, 1,158)
Random Effects	(%CV)	%RSE	95% CI	Shrinkage (%)	Bootstrap Median	Bootstrap 95% CI
IIV on E_max_	39	12	(0.080, 0.22)	29	15.50%	(0.10, 0.28)
IIV on CL	31	5	(0.075, 0.111)	12	9.50%	(0.79, 0.115)
IIV on V_1_	27	15	(0.031, 0.118)	38	6.70%	(0.44, 0.116)
Correlation CL and V_1_	39	11	(0.018, 0.047)		3.20%	(0.20, 0.047)
Residual Error	(%CV)	%RSE	95% CI			Bootstrap 95% CI
Proportional error	19	4	(0.0308, 0.0423)	8		(-0.54, -0.35)

$$CL={CL}_{TV}*e^{\frac{{E_{\max}*Time}^{Gamma}}{{T_{50}}^{Gamma}+{Time}^{Gamma}}}*{CL}_{ADA}\left(if\;ADA\;positive\right)*{\left(\frac{BLDH}{199}\right)}^{{CL}_{LDH}}*$$


$${CL}_{female}\left(iffemale\right)*{\left(\frac{BALB}{43.7}\right)}^{{CL}_{Albumin}}*{\left(\frac{BWT}{64}\right)}^{{CL}_{Weight}}*{\left(\frac{CRCL}{94.31}\right)}^{{CL}_{CRCL}}$$


$$V1={V1}_{TV}*{V1}_{White\;Race}\left(if\;White\;Race\right)*{V1}_{Other\;Race}\left(if\;Other\;Race\right)*{\left(\frac{BWT}{64}\right)}^{{V1}_{Weight}}$$

Abbreviations: %CV = percentage of the coefficient of variation; %RSE = percentage of the relative standard error; ADA = antidrug antibody; BLDH = baseline lactate dehydrogenase; BALB: baseline albumin; BWT = baseline body weight; CI = confidence interval; CL = clearance; CRCL = creatinine clearance; E_max_ = maximum effect (maximum effect of time-varying CL in log form; exponentiated value of exp [−0.444] is 0.64); Gamma = sigmoidicity of the relationship with time (T) after first dose in sigmoidal-E_max_ model for CL; IIV = interindividual variability; LDH = lactate dehydrogenase; PK = pharmacokinetic; Q = intercompartmental clearance; T_50_ = time (h) at which the change in CL is 50% of E_max_; TV = typical value; V_1_ = volume of distribution of the central compartment; V_2_ = volume of distribution of the peripheral compartment.

#### Model qualification

Bootstrap analysis was used to evaluate the stability and performance of the final PopPK model. Based on the bootstrap analysis, 909 successful minimization models of the 1,000 replicates were used to derive the 95% CI in the parameter estimate in the final model. The final PopPK model parameter estimates were consistent with the 95% CI determined by a bootstrap analysis (Table 1).

**FIGURE 1:**
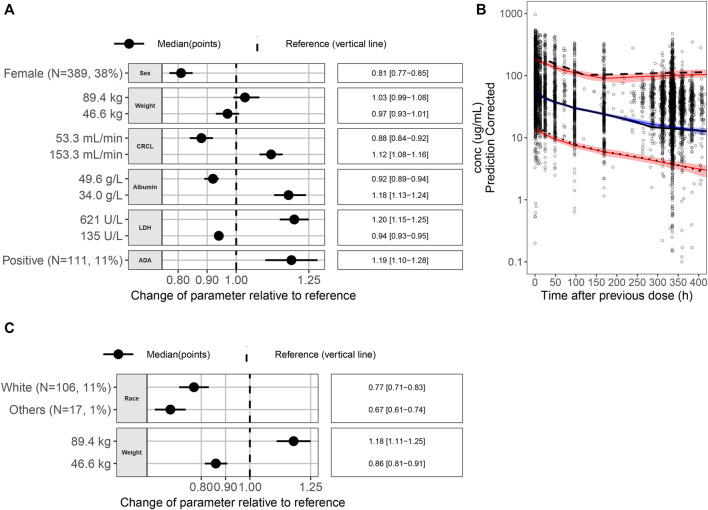
**(A)** Covariate effects on CL. **(B)** pcVPC for the final PopPK model. Black dots are the observed data points. The black solid line is the observed median; the black dashed line is the observed p95; and the black dotted line is the observed p5. The blue solid line is the simulated median, and the red solid lines are simulated p5 and p95. The blue area is the 95% PI of the simulated median, and the pink areas are the 95% PI of the simulated p5 and p95. **(C)** Covariate effects on V_1_. Abbreviations: p5 = 5th percentile; p95 = 95th percentile; pcVPC = prediction-corrected visual predictive check; PI = prediction interval; PK = pharmacokinetic; ADA = antidrug antibody; CI = confidence interval; CL = clearance; CRCL = creatinine clearance; LDH = lactate dehydrogenase; N = number of patients; V_1_ = volume of distribution.

A prediction-corrected VPC (pcVPC) for the PopPK model was performed (Figure 1B). The final PopPK model could predict the observed median, 5th percentile (p5), and 95th percentile (p95) concentration–time profiles with good agreement in the time course to 2 weeks (336 h, the nominal dosing interval) after a previous dose. Overall, the plots indicate that the final model could predict the observed toripalimab concentrations reasonably well for both the median and 95% CI of observations.

#### Assessing clinical relevance of covariates

Figures 1A,C show the impact of individual covariates on CL and V_1_ parameters, respectively. Forest plots were generated to show the p5 and p95 of a covariate and its impact on the PK parameters CL and V_1_ for a typical patient with the following characteristics: race, Asian; body weight, 63.5 kg; sex, male; ALB level, 43.6 g/L; LDH level, 198 U/L; CRCL rate, 94.3 mL/min, and ADA status, negative. The covariates were not considered clinically relevant if the effect size of each covariate on the associated PK parameter was within 80%–125% of the PK parameter value of the reference subject. In this study, the reference body weight was 63.5 kg, and the 5th percentile and 95th percentile of weight were 46.6 kg and 89.4 kg. As seen from Figures 1A,C, compared with the reference patient, the impact [median (95% confidence interval)] of 46.6 kg weight on the PK parameters CL and V1 was 0.97 (0.93–1.01) and 0.86 (0.81–0.91) compared with reference patient, respectively, and similarly, the impact of 89.4 kg weight on the PK parameters CL and V1 was 1.03 (0.99–1.08) and 1.18 (1.11–1.25). The effect sizes of 46.6 kg and 89.4 kg weights on the CL and V1 were within 80%–125% of the CL and V1 of the reference subject. Therefore, the body weight may have no clinical relevance to justify dose modifications.

#### Exposure comparison of toripalimab at 240 mg Q3W and 3 mg/kg Q2W

**FIGURE 2:**
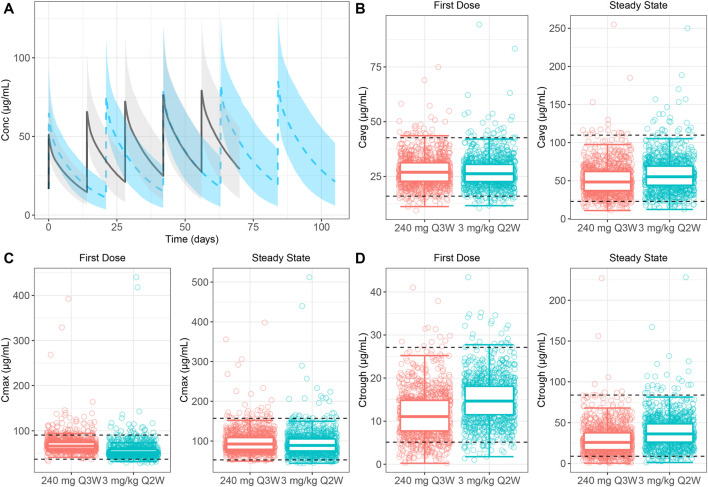
**(A)** Simulated concentration–time profiles of five doses for 3 mg/kg Q2W and 240 mg Q3W dosing regimens. The 3 mg/kg Q2W dosing regimen is represented by black lines (median) and grey shading (95% confidence interval). The 240 mg Q3W dosing regimen is represented by dashed blue lines (median) and blue shading (95% confidence interval). **(B–D) (B)** C_avg_, **(C)** C_max_ and **(D)** C_trough_ of 240 mg Q3W contained within the 2.5th and 97.5th percentiles of 3 mg/kg Q2W at the first dose and steady state. Horizontal dashed lines represent the 2.5th and 97.5th percentiles of 3 mg/kg Q2W exposures. For the first dose and steady state of C_avg_, 95% of exposure from dosing 240 mg Q3W is contained within the horizontal lines. For the first dose and steady state of C_max_, 92% and 96% of exposures from dosing 240 mg Q3W are contained within the horizontal lines, respectively. For the first dose and steady state of C_trough_, 88% and 92% of exposures from dosing 240 mg Q3W are contained within the horizontal lines, respectively. Abbreviations: C_avg_ = average serum concentration–time curve over the dosing interval; C_max_ = maximum serum concentration; C_trough_ = trough serum concentration; Q2W = every 2 weeks; Q3W = every 3 weeks.

The relative exposure between 240 mg Q3W and 3 mg/kg Q2W dosing regimens was compared by calculating the difference of the log-transformed values for each parameter for each individual. The mean difference was computed across all simulated subjects, and 90% CI was calculated. The mean difference and CIs were back-transformed to the normal scale and presented as a decimal percent relative to the 240 mg Q3W dosing regimen value in Table 2. The relative exposure estimates for all PK parameters ranged from 0.795 to 0.797. A plot of the simulated concentration–time profiles for the two dosing regimens is shown in Figure 2A. This suggests that the 3 mg/kg Q2W dosing regimen provides ∼80% of the exposure as the 240 mg Q3W dosing regimen after the first dose and at SS.

**TABLE 2:** Exposure comparison of toripalimab at 240 mg Q3W and 3 mg/kg Q2W.

Exposure	240 mg Q3W	3 mg/kg Q2W	Relative exposure
AUC_0-τ_ Dose 1 (h•µg/mL)	13386 (27) [4,309, 38539]	8,894 (26.7) [3,201, 32952]	0.796 [0.787, 0.804]
C_avg_ Dose 1 (μg/mL)	26.7 (25.2) [9.4, 75]	26.3 (25.1) [10.5, 94.3]	0.796 [0.787, 0.804]
C_max_ Dose 1 (µg/mL)	67.1 (21) [32.5, 392.5]	53.9 (23.7) [28.1, 440.1]	0.797 [0.789, 0.805]
C_trough_ Dose 1 (μg/mL)	10.2 (61.8) [0.2, 41]	13.9 (43.5) [1, 43.4]	0.795 [0.787, 0.803]
AUC_0-τ_ SS (h•µg/mL)	25555 (43.6) [5,422, 120413]	19644 (41.6) [4,067, 75932]	0.795 [0.787, 0.804]
C_avg_ SS (μg/mL)	50.7 (43.6) [10.8, 238.9]	58.5 (41.6) [12.1, 226]	0.795 [0.787, 0.804]
C_max_ SS (µg/mL)	97.6 (26.4) [50.4, 397.2]	93.7 (28.9) [43, 532.2]	0.796 [0.788, 0.805]
C_trough_ SS (μg/mL)	26.3 (85.5) [0.3, 218.5]	38.1 (65.1) [1.2, 212.6]	0.795 [0.787, 0.803]

Values for the two dose levels (240 mg Q3W and 3 mg/kg Q2W) are reported as geometric mean in µg/mL (%CV) [minimum, maximum]; Values for the relative exposure are reported as geometric mean of the ratio of 3 mg/kg Q2W to 240 mg Q3W and [lower 90% confidence interval, upper 90% confidence interval]; Dose 1 indicates first dose and SS indicates steady state.

Abbreviations: %CV = percentage of the coefficient of variation; AUC_0-τ_ = area under the serum concentration–time curve over the dosing interval; C_avg_ = average serum concentration–time curve over dosing interval; C_max_ = maximum serum concentration; C_trough_ = trough serum concentration; Q2W = every 2 weeks; Q3W = every 3 weeks

Simulations for the first dose and SS exposures for the 240 mg Q3W dosing regimen were comparable to those for the 3 mg/kg Q2W dosing regimen, with 95% exposure coverage ranging from 88% to 96%. Quantile boxplots for C_avg_, C_max_, and C_trough_ illustrating exposure coverage for 240 mg Q3W to 3 mg/kg Q2W dosing regimens are provided in Figure 2B, Figure 2C, and Figure 2D. In terms of C_avg_, C_max_, and C_trough_, the exposure to the 240 mg Q3W dosing regimen was 95%, 92%, and 88% comparable to the exposure to the 3 mg/kg Q2W dosing regimen for the first dose and 95%, 96%, and 92% comparable to the exposure to the 3 mg/kg Q2W dosing regimen at SS with 95% coverage.

### E-R analysis

#### Safety evaluation of toripalimab 240 mg Q3W

**FIGURE 3:**
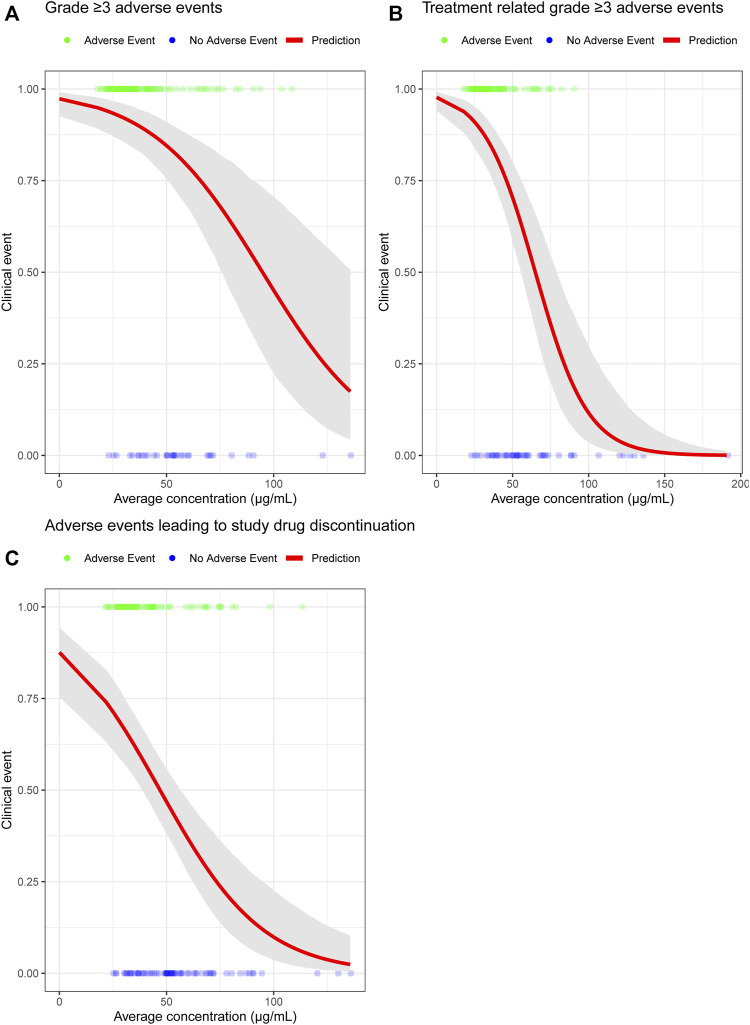
**(A)** Probability of grade ≥ 3 AEs exposure-response model for toripalimab combination therapy. **(B)** Probability of treatment-related grade ≥ 3 AEs exposure-response model for toripalimab combination therapy. **(C)** Probability of AEs leading to study drug discontinuation exposure-response model for toripalimab combination therapy. The solid red line is the predicted probability of experiencing three different categories of adverse events. The gray-shaded area is the 95% CI of the model prediction. Abbreviation: CI = confidence interval.

Although the predicted toripalimab exposures of 240 mg Q3W and 3 mg/kg Q2W were comparable, we also analyzed the safety results of 240 mg Q3W toripalimab in the JUPITER-02 study. The safety E-R analysis dataset included 152 NPC patients from the JUPITER-02 study. The safety E-R analysis examined three different categories of AEs: grade ≥ 3 AEs, treatment-related grade ≥ 3 AEs, and AEs leading to study drug discontinuation. The incidence rates for grade ≥ 3 AEs, treatment-related grade ≥ 3 AEs, and AEs leading to study drug discontinuation were 78%, 68%, and 53%, respectively, which has been summarized in Table 3. The relationships of toripalimab C_ave_ with grade ≥ 3 AEs, treatment-related grade ≥ 3 AEs, and AEs leading to study drug discontinuation are shown in Figure 3. The probability of an adverse event did not increase with increasing toripalimab exposure.

**TABLE 3:** Summary statistics for AE categories.

	AE Category	Patients with AEs	Patients without AEs	Total patients	Incidence rate
Toripalimab in combination with chemotherapy	Grade ≥ 3 AEs	118	34	152	0.78
	Treatment-related grade ≥ 3 AEs	103	49	152	0.68
	AEs leading to study drug discontinuation	81	71	152	0.53

Several studies have shown that patients with low body weight may have a relatively high drug exposure with a flat dosing regimen. In this study, the minimum weight of patients in the 240 mg Q3W dose group was 42 kg in the JUPITER-02 study, and a 240 mg Q3W dosing regimen is approximately equal to a 6-mg/kg dosing regimen. Toripalimab is reportedly well tolerated up to 10 mg/kg Q2W with monotherapy in early phase I studies (Wei et al., 2020). Therefore, 240 mg Q3W dose is safe for patients with low body weight.

#### ORR and PFS analysis

**FIGURE 4:**
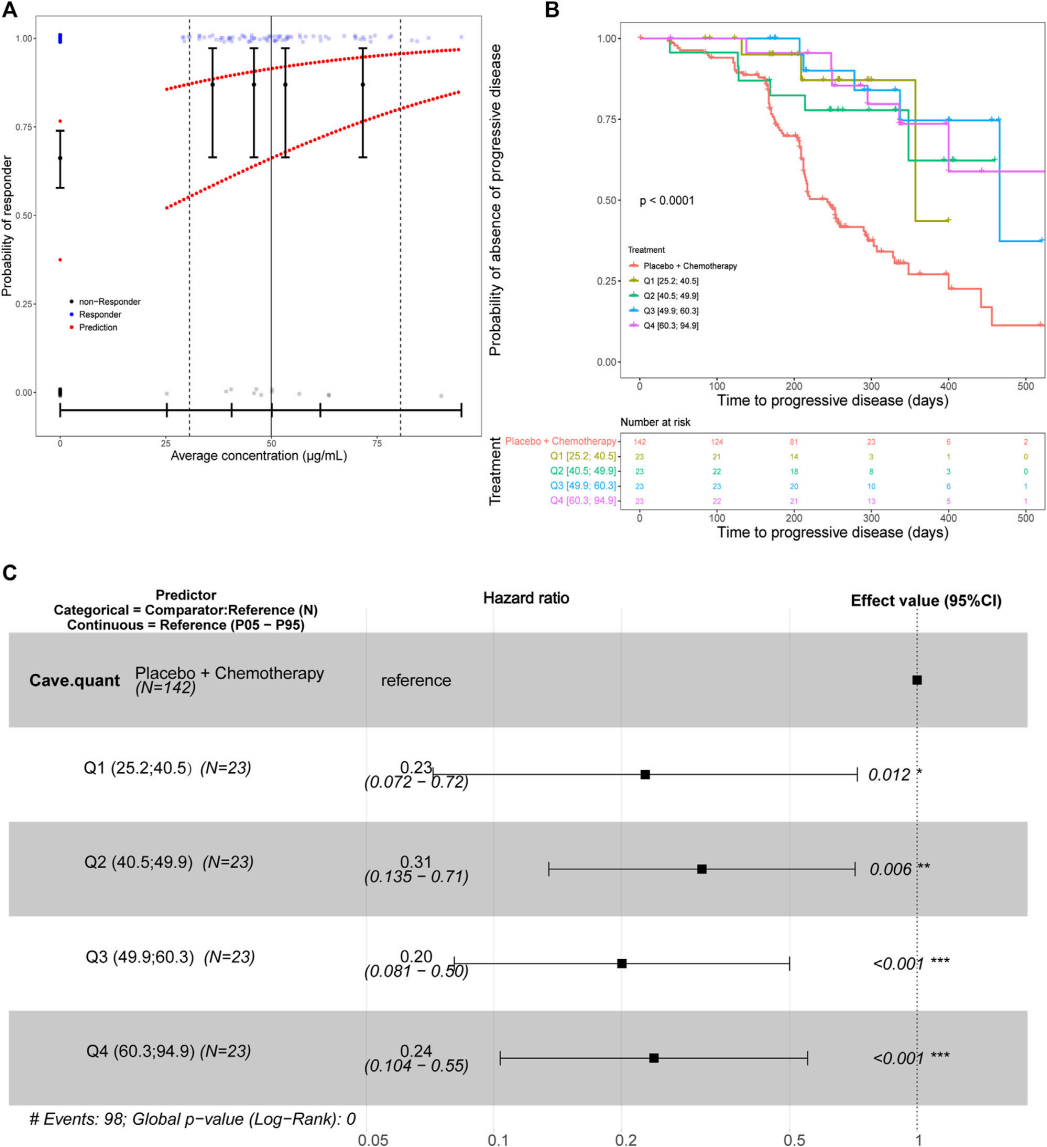
**(A)** Predicted probability of response for the final efficacy exposure-response model for the best overall response with C_ave_ as the predictor. The black circles in the error bars represent the proportion of patients who were responders at the median of the C_ave_ quartiles. Solid vertical black lines in the error bars are the 95% CIs around the probability of being a responder. The upper line of red dots is the probability for a typical patient to be a responder. The lower line of red dots is the probability for a patient with liver disease and an ECOG performance status of 1 to be a responder. The solid vertical black line is the median observed C_ave_. The dashed vertical black lines are the observed 5th and 95th C_ave_ percentiles for treated patients. The small blue and gray dots are the individual observations (jittered to more easily visualize individual patients). **(B)** The Kaplan–Meier survival curve for progression-free survival stratified by average concentration quantiles. The placebo group was considered separate from patients that received toripalimab. Therefore, the exposure quantiles are based only on the patients who received toripalimab. The number of patients at selected timepoints for each treatment group are shown in the lower panel titled “Number at risk”. **(C)** A forest plot of predictors in the progression-free survival exposure-response model with average concentration quantiles as the predictor. Abbreviations: C_ave_ = average concentration; CI = confidence interval; ECOG = Eastern Cooperative Oncology Group. N = number of patients; Q1 = first C_ave_ quartile (25.2–40.5 μg/mL); Q2 = second C_ave_ quartile (40.5–49.9 μg/mL); Q3 = third C_ave_ quartile (49.9–60.3 μg/mL); Q4 = fourth C_ave_ quartile (60.3–94.9 μg/mL); P05 = 5th percentile; P95 = 95th percentile.

E-R for efficacy was evaluated for multiple endpoints, including ORR and PFS in the JUPITER-02 study, when toripalimab was administered in combination with gemcitabine and cisplatin. For ORR efficacy analysis, a flat trend between the response rate and average concentration was observed (Figure 4A). On PFS efficacy analysis, the C_ave_ quantile was identified as a significant predictor (Figure 4B) and was included in the Cox proportional hazards regression model. Forest plots of the hazard ratios are provided in Figure 4C. Hazard ratios for exposure were significantly different from 1 (*p* < 0.001), with point estimates of risk reductions (1—hazard ratio) being approximately 70%. This has demonstrated that the risk reduction is constant across C_ave_ quartiles and risk reduction is maximized. In addition, the exposures in the lowest quartile (25.2–40.5 μg/mL) was approximately eight-fold higher than the concentration required to ensure full receptor occupancy in peripheral blood mononuclear cell target concentration (>3 μg/mL, Supplementary Figure S1). The flat E-R relationship of ORR and constant risk reduction across C_ave_ quantiles suggest that toripalimab efficacy is the highest in the 240 mg Q3W dosing regimen.

In summary, flat dosing regimen of 240 mg Q3W was demonstrated as an alternative dosing regimen of toripalimab, which was based on the predicted similar toripalimab exposures between 240 mg Q3W and the initially approved 3 m/kg Q2W regimen, the safety profile of toripalimab up to 10-mg/kg dose level, and the relatively flat safety and efficacy E-R results of 240 mg Q3W flat dosing regimen in the JUPITER-02 study.

## Discussion

In this study, a final PopPK model was established for toripalimab as monotherapy or in combination with chemotherapy on the basis of 10,430 PK observations obtained from 1,014 patients across 13 clinical studies and multiple tumor types. The final model used for the analysis of this dataset was a two-compartment model with zero-order IV infusion and time-varying CL characterized by a sigmoidal maximum effect (E_max_) function. Covariate analysis was explored to further explain PK parameter variability. Covariates from the PK analysis were included in the final model and included the impact of body weight, ALB, LDH, CRCL, sex, and the ADA-positive status on CL as well as race and body weight on V_1_. Several standard diagnostic plots were used in model development to ensure that the final model adequately describes the observed data. Tumor type and renal or hepatic impairment not being significant covariates suggests that the PopPK model can estimate drug exposure in patients with various tumors or renal or hepatic dysfunction. Similar findings were observed for pembrolizumab and nivolumab. It has been approved by the FDA that dose adjustment is not required in patients with hepatocellular carcinoma or renal cell carcinoma for pembrolizumab or nivolumab (FDA, 2022a; FDA, 2022b).

PK exposure metrics were compared between 240 mg Q3W and 3 mg/kg Q2W dosing regimens based on *post-hoc* compartmental PK parameters for the first dose and at SS. Simulations for the first dose and SS exposure for the 240 mg Q3W dosing regimen are predicted to achieve generally comparable outcomes as the 3 mg/kg Q2W dosing regimen and are within 95% exposure coverage ranging from 88% to 96%. C_trough_ at SS for 240 mg Q3W and 3 mg/kg Q2W dosing regimens was 26.3 μg/mL and 38.1 μg/mL, respectively. This change in toripalimab minimum concentration is less likely to impact the full receptor occupancy of PD-1 receptors on T cells because the simulated C_trough_ of the proposed dosing regimens substantially exceeded the target concentration (>3 μg/mL) required for full engagement of PD-1 receptors *in vitro* experiments (Supplementary Figure S1). These results are in agreement with previous findings that overall PK exposures with flat and weight -based dosing are comparable for immuno-oncology agents, including nivolumab 240 mg Q2W and 3 mg/kg Q2W (Zhao et al., 2017), pembrolizumab 200 mg Q3W and 2 mg/kg Q3W (Freshwater et al., 2017), and durvalumab 1,500 mg Q4W or 750 mg Q2W and 10 mg/kg Q2W (Baverel et al., 2018).

The model-based analysis projected drug exposure to bridge clinical safety and efficacy of the toripalimab 240 mg Q3W dosing regimen to 3 mg/kg Q2W dosing regimen. We used C_ave_ estimations to inform the safety and efficacy of the dosing regimens. Regarding safety, immune checkpoint antibodies are well-tolerated and have a manageable safety profile with a relatively low incidence of toxicity. The toripalimab dosing regimen was well tolerated with a wide dose range (1–10 mg/kg) in patients treated with monotherapy (Wei et al., 2020; Yang et al., 2020). For instance, the incidence of treatment-related AEs leading to study drug discontinuation was 11% in 862 heterogeneous patients with advanced solid tumors or lymphoma (Supplementary Table S1). In toripalimab combination therapy (JUPITER-2), the incidence of treatment-related AEs leading to study drug (toripalimab or placebo) dose interruption was only marginally higher in the toripalimab treatment arm than in the placebo arm [35.8% in the toripalimab + paclitaxel and cisplatin (TP) group vs. 25.7% in the placebo + TP group, data not published before]. Considering that 25.7% of the incidence of treatment-related AEs leading to study drug interruption in JUPITER-2 was attributed to chemotherapy, toripalimab was responsible for only the remaining 10.1% of treatment-related AEs leading to study drug interruption. E-R analyses with safety endpoints indicate that the incidence of grade ≥ 3 AEs, treatment-related grade ≥ 3 AEs, and AEs leading to study drug discontinuation does not increase with increasing toripalimab exposure in patients with NPC undergoing combinational therapy. These safety profiles are consistent with previous observations in monotherapy studies that included 12 pooled clinical studies (seven phase I, one phase Ib/II, and four phase II studies) investigated predominantly in China (Study TAB001-01 was investigated in the US; Supplementary Figure S2). Nevertheless, no consistent increase in the incidence or severity of chemotherapy-related treatment-emergent AEs was noted. The reasons for the decreased trend of incidence of grade ≥ 3 AEs, treatment-related grade ≥ 3 AEs, and AEs leading to study drug discontinuation despite increased toripalimab dosage remain unclear; however, it may be attributed to the duration of toripalimab treatment and the incidence of AEs. Patients who continue treatment and do not drop-off early because of an adverse event or disease progression are less likely to experience a clinically meaningful adverse event. The longer the patients stayed with the treatment, the higher was the concentration accumulated due to the time-dependent decreases in CL.

Although E-R datasets were constructed using data at a single-dose level, the analyses included a wide range of exposures (9.9–110 μg/mL). This exposure range enables a meaningful evaluation of E-R relationships. Efficacy E-R analysis based on ORR or PFS included the available PK data from patients in both arms of the JUPITER-02 study. A flat E-R relationship with efficacy was identified over this dose range. The ORR is not dependent on the average concentration. Cox proportional hazards model-based analyses for PFS suggest that risk reduction is constant across concentration quartiles. The treatment (toripalimab 240 mg in combination with chemotherapy) offers an approximately 70% reduced risk of disease progression over placebo plus chemotherapy. The constant risk reduction across C_ave_ quantiles and the flat E-R relationship of ORR suggest toripalimab efficacy is maximum in the 240 mg Q3W dosing regimen. At the respective approved dosages, pembrolizumab and nivolumab achieve full PD-1 receptor engagement, and efficacy is generally associated with the flat portion of the E-R curve across indications as well (Pluim et al., 2019).

Although weight-based dosing tends to yield the desired minimization in interindividual variation in exposure, flat dosing did not lead to significantly different interindividual pharmacokinetic variability in drug exposure (Smorenburg et al., 2003; de Jong et al., 2004). Model development has revealed that body weight anticipates <20% variability of V_1_ when compared to a hypothetical reference patient. This variability may not result in significant changes in either efficacy or safety response because of the wide therapeutic index and the flat E-R relationship of toripalimab. When using a weight-based dosing regimen, the contents of drug ampules are typically incompletely administered. Owing to its greater simplicity, flexibility, and convenience, flat dosing is perhaps superior to weight-based regimens for both patients and healthcare providers in terms of the optimal therapeutic use of anti-PD-1 antibodies. It would improve medication compliance and may also reduce the risk of medication errors by reducing dosing complexity associated with the varying number of visits to treatment centers and medication dose calculations. In the current analyses, flat dose regimens of toripalimab showed comparable efficacy and safety profiles as weight-based dosing regimens while offering greater flexibility and convenience with the treatment. Notably, 240-mg toripalimab administered in combination with chemotherapy drugs intravenously every 3 weeks matches a chemotherapy cycle for many antitumor drugs.

Moreover, flat dosing of toripalimab may be more economical than weight-based dosing. Toripalimab has been available as 80 mg/2 mL and 240 mg/6 mL solutions in a single-dose vial in China. The price of one vial of 240-mg is almost the same as the price of three vials of 80-mg. In this PopPK analysis of toripalimab, the patients had a mean weight of 65 kg (range, 31.6–164 kg). For patients weighing 31.6–53.3 kg, 12 vials of 80-mg toripalimab or 4 vials of 240-mg toripalimab would be required every three months with a weight-based or flat dosing regimen, and the drug cost of the two regimens would be the same. However, patients weighing more than 53.3 kg need more than 12 vials of 80-mg toripalimab every three months with the weight-based regimen, and thus, flat dosing regimen seems more economical than weight-based dosing regimen in such patients. In addition, patients treated once every 3 weeks will have fewer visits to hospitals or clinics, thus reducing the corresponding medicare costs.

There are still some limitations in our study. Some factors like PK sample collection, sample size, study design may be potential sources of bias and imprecision of PopPK model. This PopPK model will be updated and optimized with more and more clinical researches in the future.

The results presented in this study demonstrate that the toripalimab 240 mg Q3W dosing regimen achieves exposures comparable to the toripalimab 3 mg/kg Q2W dosing regimen. Moreover, flat dosage can be considered an appropriate dose strategy for toripalimab as it reaches exposures well above the concentration required for full receptor occupancy as shown in JUPITER-02. Furthermore, it has also been demonstrated to achieve near maximal efficacy and have acceptable tolerability. Additionally, the 240-mg Q3W dose of toripalimab, which is currently being investigated in combination with different therapies for various oncology indications, provides a convenient dose strategy with a proven therapeutic efficacy and a tolerable safety profile.

## Data Availability

The original contributions presented in the study are included in the article/Supplementary Material, further inquiries can be directed to the corresponding author.

## Supplementary material

Supplementary Figure S1 Toripalimab binds with PD-1 expressed on the surface of human activated T cells. Note: Y-axis represents the percentage of receptor occupancy on activated CD4+ and CD8+ T cells in four donors. X-axis represents a series of toripalimab concentrations. Supplementary Figure S1 **(A)**,**(B)**, **(C)**, and **(D)** represent four different donors. Abbreviations: EC50, half-maximal effective concentration.

Supplementary Figure S2 Probability of adverse event exposure-response model for toripalimab monotherapy. The solid red line is the predicted probability of experiencing three different categories of adverse events [grade ≥ 3 AEs, treatment-related grade ≥ 3 AEs, and AEs leading to study drug discontinuation]. The gray-shaded area is the 95% CI of the model prediction. Abbreviation: CI = confidence interval.

## Glossary

AUC: area under the curve
ADA: anti-drug antibody
AE: adverse events
BALB: baseline albumin
BAST: baseline aspartate aminotransferase
BALT: baseline alanine aminotransferase
BLDH: baseline lactate dehydrogenase
BILI: baseline total bilirubin
BWT: baseline body weight
C_max_: maximum serum concentration
C_avg_: average serum concentration–time curve over the dosing interval
C_ave_: average concentration
C_trough_: trough serum concentration
CL: clearance
COMBOTRT: combination treatment
CRCL: creatinine clearance
E_max_: sigmoidal maximum effect
ECOG: Eastern Cooperative Oncology Group
ESCC: esophageal squamous cell carcinoma
E-R analysis: exposure-response analysis
FDA: Food and Drug Administration
GC: gastric cancer
KIDIMBLN: Kidney impairment at baseline
LVRIMBLN: liver impairment at baseline
mAb: monoclonal antibody
NONMEM: non-linear mixed-effects modeling
NPC: nasopharyngeal carcinoma
NSCLC: non-squamous non-small cell lung cancer
OFV: lower objective function value
PD-1: programmed death receptor-1
PD-L1: programmed death-ligand 1
PFS: progression-free survival
RP2D: recommended phase II dose
SCM: stepwise covariate model
TUBURB: baseline tumor burden
UC: urothelial carcinoma
V_1_: central volume of distribution
VPC: visual predictive check

## References

[B1] Bai S. Jorga K. Xin Y. Jin D. Zheng Y. Damico-Beyer L. A. (2012). A guide to rational dosing of monoclonal antibodies. Clin. Pharmacokinet. 51 (2), 119–135. 10.2165/11596370-000000000-00000 22257150

[B2] Baverel P. G. Dubois V. F. S. Jin C. Y. Zheng Y. Song X. Jin X. (2018). Population pharmacokinetics of durvalumab in cancer patients and association with longitudinal biomarkers of disease status. Clin. Pharmacol. Ther. 103 (4), 631–642. 10.1002/cpt.982 29243223 PMC5887840

[B3] Bi Y. Liu J. Furmanski B. Zhao H. Yu J. Osgood C. (2019). Model-informed drug development approach supporting approval of the 4-week (Q4W) dosing schedule for nivolumab (opdivo) across multiple indications: A regulatory perspective. Ann. Oncol. 30 (4), 644–651. 10.1093/annonc/mdz037 30715147

[B4] de Jong F. A. Mathijssen R. H. Xie R. Verweij J. Sparreboom A. (2004). Flat-fixed dosing of irinotecan: Influence on pharmacokinetic and pharmacodynamic variability. Clin. Cancer Res. 10 (12 Pt 1), 4068–4071. 10.1158/1078-0432.ccr-03-0591 15217940

[B5] FDA (2022a). Shanghai Junshi Biosciences Co., Ltd. Shanghai: Toripalimab. https://www.junshipharma.com/en/rd-pipeline/ (Accessed January 3, 2023)

[B6] FDA (2022b). US Food and Drug Administration. Washington DC: KEYTRUDA. https://www.accessdata.fda.gov/drugsatfda_docs/label/2022/125514s127lbl.pdf (Accessed January 3, 2023)

[B7] Freshwater T. Kondic A. Ahamadi M. Li C. H. de Greef R. de Alwis D. (2017). Evaluation of dosing strategy for pembrolizumab for oncology indications. J. Immunother. Cancer 16 (5), 43. 10.1186/s40425-017-0242-5 PMC5433037 28515943

[B8] Hendrikx J. Haanen J. Voest E. E. Schellens J. H. M. Huitema A. D. R. Beijnen J. H. (2017). Fixed dosing of monoclonal antibodies in oncology. Oncologist 22 (10), 1212–1221. 10.1634/theoncologist.2017-0167 28754722 PMC5634778

[B9] Keam S. J. (2019). Toripalimab: First global approval. Drugs 79 (5), 573–578. 10.1007/s40265-019-01076-2 30805896

[B10] Mai H. Q. Chen Q. Y. Chen D. Hu C. Yang K. Wen J. (2022). Abstract CT226: Final progression-free survival analysis of JUPITER-02, a randomized, double-blind, phase 3 study of toripalimab or placebo plus gemcitabine and cisplatin as first-line treatment for recurrent or metastatic nasopharyngeal carcinoma. Cancer Res. 82 (12 _Suppl. ment), CT226. 10.1158/1538-7445.AM2022-CT226

[B11] Mai H. Q. Chen Q. Y. Chen D. Hu C. Yang K. Wen J. (2021). Toripalimab or placebo plus chemotherapy as first-line treatment in advanced nasopharyngeal carcinoma: A multicenter randomized phase 3 trial. Nat. Med. 27 (9), 1536–1543. 10.1038/s41591-021-01444-0 34341578

[B12] Patsoukis N. Wang Q. Strauss L. Boussiotis V. A. (2020). Revisiting the PD-1 pathway. Sci. Adv. 6 (38), eabd2712. 10.1126/sciadv.abd2712 32948597 PMC7500922

[B13] Pluim D. Ros W. Miedema I. H. C. Beijnen J. H. Schellens J. H. M. (2019). Multiparameter flow cytometry assay for quantification of immune cell subsets, PD-1 expression levels and PD-1 receptor occupancy by nivolumab and pembrolizumab. Cytom. A 95 (10), 1053–1065. 10.1002/cyto.a.23873 31407460

[B14] Sheng X. Chen H. Hu B. Yao X. Liu Z. Yao X. (2022). Safety, efficacy, and biomarker analysis of toripalimab in patients with previously treated advanced urothelial carcinoma: Results from a multicenter phase II trial POLARIS-03. Clin. Cancer Res. 28 (3), 489–497. 10.1158/1078-0432.ccr-21-2210 34740921

[B15] Smorenburg C. H. Sparreboom A. Bontenbal M. Stoter G. Nooter K. Verweij J. (2003). Randomized cross-over evaluation of body-surface area-based dosing versus flat-fixed dosing of paclitaxel. J. Clin. Oncol. 21 (2), 197–202. 10.1200/jco.2003.01.058 12525510

[B16] Tang B. Chi Z. Chen Y. Liu X. Wu D. Chen J. (2020). Safety, efficacy, and biomarker analysis of toripalimab in previously treated advanced melanoma: Results of the POLARIS-01 multicenter phase II trial. Clin. Cancer Res. 26 (16), 4250–4259. 10.1158/1078-0432.ccr-19-3922 32321714

[B17] Wang F. H. Wei X. L. Feng J. Li Q. Xu N. Hu X. C. (2021). Efficacy, safety, and correlative biomarkers of toripalimab in previously treated recurrent or metastatic nasopharyngeal carcinoma: A phase II clinical trial (POLARIS-02). J. Clin. Oncol. 39 (7), 704–712. 10.1200/jco.20.02712 33492986 PMC8078488

[B18] Wang J. Wang Z. Wu L. Li B. Cheng Y. Li X. (2022a). Final progression-free survival, interim overall survival, and biomarker analyses of CHOICE-01: A phase 3 study of toripalimab versus placebo in combination with first-line chemotherapy for advanced NSCLC without EGFR/ALK mutations. ASCO Mon. Plenary Ser., 40, (16 _suppl).

[B19] Wang Y. Zhu H. Madabushi R. Liu Q. Huang S. M. Zineh I. (2020). Model-informed drug development: Current US regulatory practice and future considerations. Clin. Pharmacol. Ther. 105 (4), 899–911. 10.1002/cpt.1363 30653670

[B20] Wang Z. Wu L. Li B. Cheng Y. Li X. Wang X. (2022b). Toripalimab plus chemotherapy for patients with treatment-naive advanced non-small-cell lung cancer: A multicenter randomized phase III trial (CHOICE-01). J. Clin. Oncol., JCO2200727. 10.1200/jco.22.00727 PMC9870236 36206498

[B21] Wang Z. X. Cui C. Yao J. Zhang Y. Li M. Feng J. (2022c). Toripalimab plus chemotherapy in treatment-naïve, advanced esophageal squamous cell carcinoma (JUPITER-06): A multi-center phase 3 trial. Cancer Cell 40 (3), 277–288.e3. 10.1016/j.ccell.2022.02.007 e273 35245446

[B22] Wei X. L. Ren C. Wang F. H. Zhang Y. Zhao H. Y. Zou B. Y. (2020). A phase I study of toripalimab, an anti-PD-1 antibody, in patients with refractory malignant solid tumors. Cancer Commun. (Lond) 40 (8), 345–354. 10.1002/cac2.12068 32589350 PMC7427305

[B23] Xu R. H. Mai H. Q. Chen Q. Y. Chen D. Hu C. Yang K. (2021a). JUPITER-02: Randomized, double-blind, phase III study of toripalimab or placebo plus gemcitabine and cisplatin as first-line treatment for recurrent or metastatic nasopharyngeal carcinoma (NPC). J. Clin. Oncol. 39. 10.1200/JCO.2021.39.15_suppl (18_suppl), LBA2, 2021 ASCO Annual Meeting IILBA2

[B24] Xu R. H. Wang F. Cui C. Yao J. Zhang Y. Wang G. (2021b). 1373MO JUPITER-06: A randomized, double-blind, phase III study of toripalimab versus placebo in combination with first-line chemotherapy for treatment naive advanced or metastatic esophageal squamous cell carcinoma (ESCC). Ann. Oncol. 32, S1041. 10.1016/j.annonc.2021.08.1482

[B25] Yang J. Dong L. Yang S. Han X. Han Y. Jiang S. (2020). Safety and clinical efficacy of toripalimab, a PD-1 mAb, in patients with advanced or recurrent malignancies in a phase I study. Eur. J. Cancer 130, 182–192. 10.1016/j.ejca.2020.01.028 32224416

[B26] Zhang J. Zhou C. Zhao Y. Mu X. Zhou J. Bao Z. (2019). MA11.06 A PII study of toripalimab, a PD-1 mAb, in combination with chemotherapy in EGFR+ advanced NSCLC patients failed to prior EGFR tki therapies. J. Thorac. Oncol. 14 (10 _Suppl. l), S292. 10.1016/j.jtho.2019.08.587

[B27] Zhao X. Suryawanshi S. Hruska M. Feng Y. Wang X. Shen J. (2017). Assessment of nivolumab benefit-risk profile of a 240-mg flat dose relative to a 3-mg/kg dosing regimen in patients with advanced tumors. Ann. Oncol. 28 (8), 2002–2008. 10.1093/annonc/mdx235 28520840 PMC5834087

