# Supplementary material for: Flat dose regimen of toripalimab based on model-informed drug development approach
Source: Front Pharmacol. 2023 Jan 13;13:1069818. doi: 10.3389/fphar.2022.1069818 (PMC9880172; doi:10.3389/fphar.2022.1069818)
Supplement: Supplementary file 1 [file Table1.DOCX]

**Table S1.** Clinical Trials of Toripalimab Included in PopPK analysis

| **Protocol Number and Title** | **Population** | **Toripalimab**  **Dose** | **Patients**  **(Excluded Patients)** | **Measurable Observations**  (Excluded Observations) |
| --- | --- | --- | --- | --- |
| **CT1:** A Phase 1, Open-label, Single-center, Dose Escalation Study Investigating the Tolerability and Pharmacokinetics of a Single Dose and Multiple Doses of Recombinant Humanized Anti‑PD‑1 Monoclonal Antibody Injection in Subjects with Advanced Tumors (NCT02836795) | Advanced melanoma or urologic cancer refractory to standard therapy | Escalation: 1, 3, or 10 mg/kg Q2W  Expansion: 1, 3, and 10 mg/kg Q2W | 36 (0) | 1073 (22) |
| **CT2:** A Phase 1a Clinical Study on the Safety, Tolerability, Pharmacokinetics and Pharmacodynamics of the Use of a Single Dose and Multiple Doses of Recombinant Humanized Anti-PD-1 Monoclonal Antibody Injection in Subjects with Advanced Solid Tumors (NCT02857166) | Advanced or recurrent solid tumors after failure of >1 prior systematic treatment | Escalation: 0.3, 1, 3, or 10 mg/kg Q2W  Expansion: 1, 3 and 10 mg/kg and 240 mg Q2W | 25 (0) | 411 (2) |
| **CT3:** A Phase 1 Clinical Study on the Safety, Tolerability, Pharmacokinetics and Pharmacodynamics of the Use of a Single Dose and Multiple Doses of Recombinant Humanized Anti-PD-1 Monoclonal Antibody Injection in Subjects with Advanced Malignancies (NCT02836834) | Advanced solid tumors or lymphoma refractory to standard therapy | Escalation: 1, 3, or 10 mg/kg Q2W  Expansion: 1, 3, and 10 mg/kg | 32 (0) | 577 (3) |
| **CT4:** An Open-label, Multi-center, Single-arm, Phase 2 Clinical Trial Evaluating the Efficacy and Safety of Recombinant Humanized Anti-PD-1 Monoclonal Antibody Injection in Subjects with Locally Advanced or Metastatic Melanoma After Failure of Standard Treatment (NCT03013101) | Locally advanced/metastatic melanoma after failure of standard of care | 3 mg/kg Q2W | 128 (9) | 1103 (9) |
| **CT5:** POLARIS-02: A Multi-Center, Open-label, Phase 1b/2 Clinical Study to Evaluate Toripalimab (JS001 or TAB001) in Subjects with Advanced Gastric Adenocarcinoma, Esophageal Squamous Cell Carcinoma, Nasopharyngeal Carcinoma, or Head and Neck Squamous Cell Carcinoma (NCT02915432) | Previously treated, locally advanced/metastatic GC, ESCC, NPC, or HNSCC | 3 mg/kg Q2W  240 mg or 360 mg Q3W for combination therapy | 253 (13) | 2716 (178) |
| **CT6:** A Phase 1 Clinical Study of the Safety, Tolerability, Pharmacokinetics and Pharmacodynamics of Multiple Doses of Recombinant Humanized Anti‑PD‑1 Monoclonal Antibody Injection in Subjects with Relapsed/Refractory Malignant Lymphoma (NCT03316144) | Lymphoma refractory to standard therapy | Escalation: 1, 3, or 10 mg/kg Q2W | 13 (0) | 345 (2) |
| **CT7:** A Phase 1 Study Investigating the Similarity of the Pharmacokinetics and Safety of a Single Dose and Parallel Comparison of Recombinant Humanized Anti-PD-1 Monoclonal Antibody Injection in Subjects with Advanced NSCLC Prior to and After a Manufacturing Process Change (NCT03301688) | Advanced non-small cell lung cancer refractory to standard therapy | 3 mg/kg Q2W | 67 (0) | 786 (0) |
| **CT8:** A Randomized, Controlled, Multi-Center Phase II Clinical Study Comparing Recombinant Humanized Anti-PD-1 Monoclonal Antibody Injection with High Dose Interferon as Adjuvant Therapy in the Treatment of Completely Resected Mucosal Melanoma (NCT03178123) | Resected mucosal melanoma | 3 mg/kg Q2W | 82 (13) | 585 (4) |
| **CT9:** A Phase 1, Open-label, Single‑center, Dose Escalation Study to Evaluate the Tolerability and Pharmacokinetics of a Single Dose and Multiple Doses of Recombinant Humanized Anti-PD-1 Monoclonal Antibody Injection in Advanced Triple‑Negative Breast Cancer (NCT02838823) | Triple-negative breast cancer refractory to standard therapy | Escalation: 1, 3, and 10 mg/kg Q2W  Expansion: 3 mg/kg Q2W | 20 (0) | 452 (1) |
| **CT12:** An Open-label, Multi-center, Single-arm, Phase 2 Clinical Trial Evaluating the Efficacy and Safety of Toripalimab Injection (Recombinant Humanized Anti-PD-1 Monoclonal Antibody Injection, JS001) in Subjects with Locally Advanced or Metastatic Bladder Urothelial Carcinoma after Failure of Standard Treatment (NCT03113266) | Locally advanced or metastatic urothelial cancer after >1 prior therapy | 3 mg/kg Q2W | 151 (15) | 960 (34) |
| **CT14:** A Phase Ib Clinical Trial Evaluating the Safety and Efficacy of Recombinant Humanized Anti-PD-1 Monoclonal Antibody Injection in Subjects with Advanced Neuroendocrine Tumors After Failure of Standard Treatment (NCT03167853) | Neuroendocrine tumor (NET) after > 1 prior therapy | 3 mg/kg Q2W | 40 (6) | 253 (2) |
| **CT15 (JUPITER-02):** Phase III Study of Comparing TORIPALIMAB INJECTION Versus Placebo Combined With Chemotherapy for Recurrent or Metastatic Nasophapyngeal Cancer (NCT03581786) | recurrent or metastatic nasopharyngeal  carcinoma | 240 mg Q3W | 94 (2) | 344 (0) |
| **TAB001-01:** A Phase 1, Multicenter, Open-Label Study to Evaluate the Safety, Tolerability, and Pharmacokinetics of TAB001 in Subjects with Advanced Malignancies (NCT03474640) | Solid tumors refractory to standard therapy  EC, gastric cancer, NET, sarcoma, NPC, HCC, CC,  MSI-H/dMMR > 1 prior therapy | 80, 240, or 480 mg Q2W  240 mg Q3W | 135 (4) | 1089 (7) |

ESCC: esophageal squamous-cell carcinoma; GC: gastric cancer; HNSCC: head and neck squamous cell carcinoma; EC: esophageal cancer; NET: neuroendocrine tumor; HCC: hepatocellular cancer; CC: cholangiocarcinoma; MSI-H: microsatellite instability-high; dMMR: deficient DNA mismatch repair; NPC: nasopharyngeal carcinoma; PD-1: programmed death receptor-1

**Table S2.** Summary Statistics for Adverse Events for toripalimab monotherapy

| AEs | Subjects with AEs | Subjects without AEs | Total subjects | Incidence rate |
| --- | --- | --- | --- | --- |
| Toxicity grade ≥ 3 | 334 | 528 | 862 | 0.39 |
| Toxicity grade ≥ 3 (treatment related) | 217 | 645 | 862 | 0.25 |
| Toxicity leading to study drug discontinuation | 95 | 767 | 862 | 0.11 |
| Abbreviation: AEs = adverse events | | | | |
