# Supplementary figures and images for: Flat dose regimen of toripalimab based on model-informed drug development approach
Source: Front Pharmacol. 2023 Jan 13;13:1069818. doi: 10.3389/fphar.2022.1069818 (PMC9880172; doi:10.3389/fphar.2022.1069818)

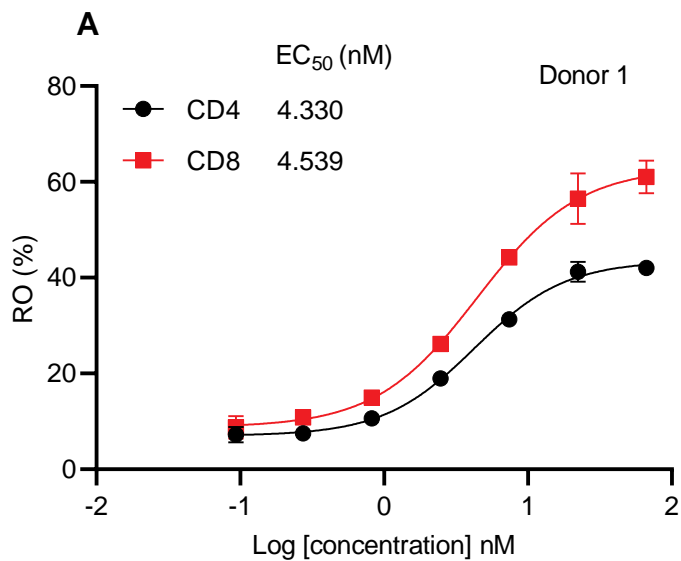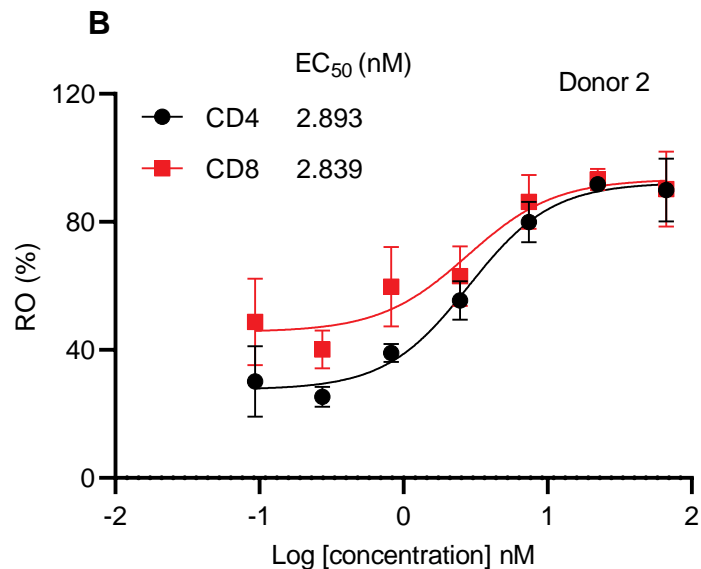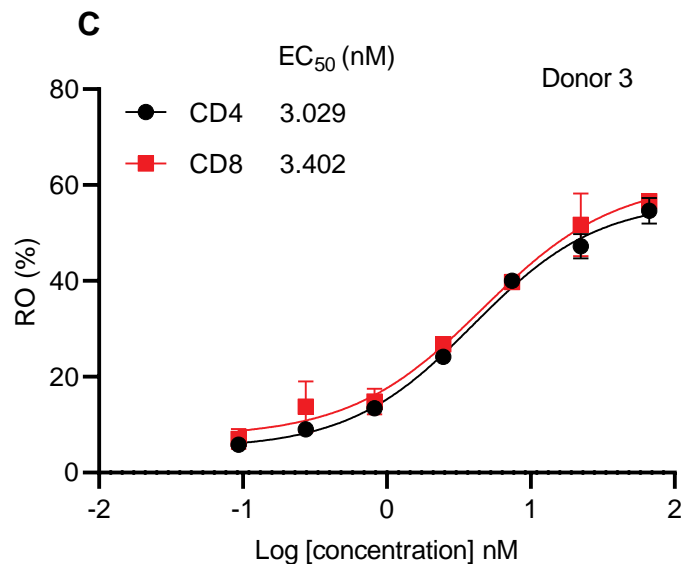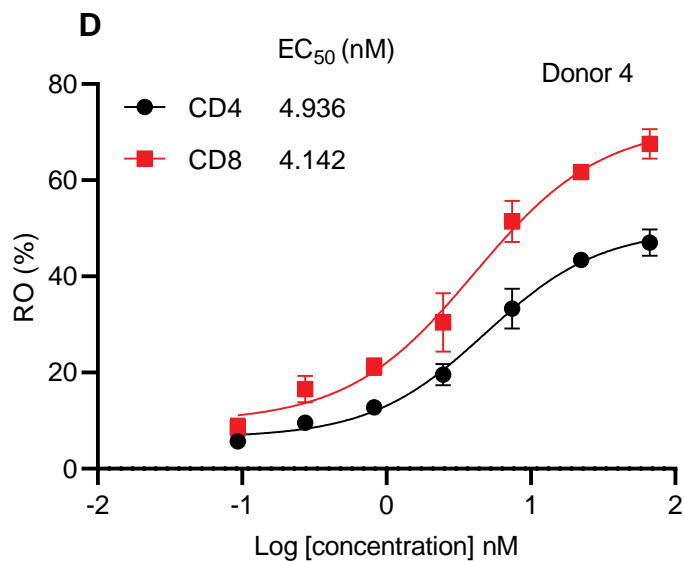

Supplement: Supplementary file 3 [file DataSheet1.PDF]
